# Evolving assessment pathways for precision oncology medicines to improve patient access: a tumor-agnostic lens

**DOI:** 10.1093/oncolo/oyae060

**Published:** 2024-04-17

**Authors:** Priscila Radu, Gayathri Kumar, Amanda Cole, Aikaterini Fameli, Mark Guthrie, Lieven Annemans, Jan Geissler, Antoine Italiano, Brian O’Rourke, Entela Xoxi, Lotte Steuten

**Affiliations:** Office of Health Economics, London, United Kingdom; Office of Health Economics, London, United Kingdom; Office of Health Economics, London, United Kingdom; Global Oncology Policy, GSK, London, United Kingdom; Global Access Strategy Oncology, Roche, San Francisco, CA, United States; Department of Health Economics, Ghent University, Ghent, Belgium; Patvocates, Munich, Germany; Early Phase Trials and Sarcoma Units, Institut Bergonié, Bordeaux, France; ISPOR, Ottawa, Canada; Faculty of Economics, ALTEMS Università Cattolica del Sacro Cuore, Rome, Italy; Office of Health Economics, London, United Kingdom

**Keywords:** precision oncology, life-cycle assessment, personalized medicine, tumor-agnostic

## Abstract

**Background:**

Genomic and molecular alterations are increasingly important in cancer diagnosis, and scientific advances are opening new treatment avenues. Precision oncology (PO) uses a patient’s genomic profile to determine optimal treatment, promising fewer side effects and higher success rates. Within PO, tumor-agnostic (TA) therapies target genomic alterations irrespective of tumor location. However, traditional value frameworks and approval pathways pose challenges which may limit patient access to PO therapies.

**Objectives:**

This study describes challenges in assessing PO and TA medicines, explores possible solutions, and provides actionable recommendations to facilitate an iterative life-cycle assessment of these medicines.

**Methods:**

After reviewing the published literature, we obtained insights from key stakeholders and European experts across a range of disciplines, through individual interviews and an industry workshop. The research was guided and refined by an international expert committee through 2 sounding board meetings.

**Results:**

The current challenges faced by PO and TA medicines are multiple and can be demonstrated through real-world examples of the current barriers and opportunities. A life-cycle approach to assessment should be taken, including key actions at the early stages of evidence generation, regulatory and reimbursement stage, as well as payment and adoption solutions that make use of the evolving evidence base. Working toward these solutions to maximize PO medicine value is a shared responsibility and stands to benefit all stakeholders.

**Conclusions:**

Our call to action is to expand access to comprehensive genomic testing, foster a learning health care system, enable fast and equitable access to cost-effective treatments, and ultimately improve health outcomes.

## Teaser

In precision oncology, tumor-agnostic therapies target genomic alterations irrespective of tumor location; however, traditional value frameworks and approval pathways can limit patient access to such therapies. This article describes challenges and possible solutions to improve patient access.

**Implications for Practice**

Scientific advances are opening new treatment avenues in oncology. However, traditional value frameworks and approval pathways pose challenges which may limit patient access. A life-cycle approach for the assessment of PO and TA drugs would help maximize their value and improve outcomes for patients by promoting dialogue between development, assessment, and access stages, and supporting a learning health care system. Embedding these solutions in the pathway is a shared responsibility that benefits all stakeholders. The aim of this call to action is to advance the dialogue and ongoing cooperation between all stakeholder communities to co-create solutions and cultivate a learning health system environment that improves health outcomes.

## Introduction

Precision oncology (PO) uses molecular, phenotypic, and health data from cancer patients to target treatment and improve health outcomes.^1^ Genomic and molecular alterations are increasingly important in cancer diagnosis, and scientific advances are opening new treatment avenues. Tumor-agnostic (TA) therapies in PO target genomic alterations in the tumor, regardless of location or tissue of origin.^2^

Precision oncology holds great promise: for better therapy selection, fewer side effects, and higher success rates,^3^ thereby having the potential to improve patient outcomes and cancer care efficiency. Furthermore, using these therapies and their associated diagnostic tools requires and therefore leads to improved data collection.^4^ Sharing and using these data could create a “learning health care system” that uses real-world evidence (RWE) to target treatment and learn about treatment outcomes, with spillover effects beyond oncology.^5^

Precision oncology holds great promise, but evidence-generation challenges and other barriers may limit patient access and increase regulatory and reimbursement decision-making uncertainty,^4^ exacerbated by limited and unequal access to diagnostic testing and treatments across jurisdictions.^3^ Tumor-agnostic therapies may have many comparators and definitions of standard of care across tumor types, which intensifies these challenges.^6^ This makes it nearly impossible to investigate interventions using traditional randomized controlled trials (RCTs); however, the acceptability of novel trial designs is variable amongst regulatory decision-makers (those ensuring a product is safe and efficacious) and health technology assessment (HTA) decision-makers (those tasked with assessing a medicine’s clinical and cost-effectiveness and making decisions about reimbursement).^7^

These challenges are a shared problem for all parties: industry developing innovative treatments, health systems trying to maximize population health from limited resources, clinicians seeking to prescribe the best therapies for their patients, and patients valuing improvements in survival and quality of life. Thus, the value of PO and TA medicines can only be realized through collaboration, with stakeholders co-owning challenges and co-creating solutions.

This study discusses PO medicine access issues and possible solutions, including TA-related factors and real-world examples. We conclude with a call to action for life-cycle assessment for PO medicines: broadening access to comprehensive molecular testing, such as next-generation sequencing (NGS) testing; working toward a learning health care system; and accelerating equitable access to the right treatments to improve patient outcomes.

## Methods

Our approach included a targeted literature search with the aim of gathering journal articles and published studies on PO benefits, policy and methodological challenges, and possible solutions. The search was supplemented by the advisors’ own knowledge of the available literature pertaining to our scope. Articles retrieved informed an initial draft of the “vision” for a PO assessment and access pathway, which guided the direction of this study.

We facilitated a workshop with the European Federation of Pharmaceutical Industries and Associations (EFPIA) Oncology Platform working group^8^ to consolidate the “vision” and leveraged their expertise to elucidate the challenges faced by these therapies. The project process and interpretation of findings were guided by a sounding board consisting of HTA, clinical oncology, and patient advocacy experts. The selection of the sounding board experts was based on their recognized expertise and experience in their respective fields, and their broad geographical and stakeholder representation. The sounding board met once before the interview phase (to refine the “vision” and direct further research), and once after (to reflect on, and further refine the revised “vision”).

We interviewed leading European experts in medical oncology, pathology, policy-making, patient advocacy, HTA, health economics, and industry representatives to identify challenges and local, national, or European initiatives. The interview process guided the participants through our initial findings and leveraged their expertise to inform and strengthen our findings and recommendations. The research focused on the UK, France, Germany, Spain, Italy, the Netherlands, Belgium, Sweden, and Canada.

## Results

### What are the challenges for PO and TA medicines?

Precision oncology medicines face various challenges throughout the life cycle (Box 1).

**Box 1.** Current challenges for PO

Early evidence generation • Small patient group sizes and heterogeneous tumor profiles • Variability in patient outcomes • Limited clinical expertise • Lack of… ◦ Long-term evidence ◦ guidance on data collection from regulators ◦ consistency in the collection of PRO ◦ well-defined (or inconsistent) comparator	Regulatory • Heterogeneity in patient characteristics and outcomes • Unclear relationship between biomarker-based endpoints and survival • Lack of… ◦ evidence for prognostic and predictive properties of biomarker ◦ consistency in collection ◦ appropriate incentives or processes to overcome clinical challenges ◦ mechanism for European Medicines Agency to identify/classify rarity for TA drugs (implications for HTA evidence requirements)
HTA • Acceptance of indirect treatment comparison • Heterogeneity in patient characteristics and outcomes • Accounting for the cost of diagnosis (genomic testing) in economic evaluations • Financial burden on patients and society	Payment and adoption • Divergent processes and systems for addressing uncertainty and reflecting this in conditional payment models • Challenges in permitting payment to vary by indication • Fragmentation of current RWD platforms • Variation in patient advocacy and understanding of disease by policymakers • Lack of access to comprehensive molecular testing • Separate budgets for implementation (eg, diagnostic test budgets compared to drug budgets) • Inability to recoup costs of single-administration therapies in Early Access Schemes • Concern that the price used in the access scheme may be the benchmark price when the drug is approved

#### Early evidence generation

Precision oncology therapies target small patient populations, and TA therapies may treat several subgroups with diverse baseline characteristics. This makes RCTs for both therapies difficult. Investigators sometimes use phase II, single-arm, or non-comparator evidence for regulatory assessment.^9,10^ Basket trials study TA therapies based on a genomic alteration, regardless of histology. These trials can more efficiently investigate efficacy across multiple patient subgroups, reducing the time needed to reach an adequately powered sample size. However, small patient sample sizes and tumor heterogeneity tend to create clinical uncertainty around treatment effect for specific tumor types or indications.^11,12^ Furthermore, basket trials and single-arm studies use response-based endpoints rather than survival endpoints, which may be challenging to incorporate in economic models, commonly used in oncology HTA. Additionally, a consistent comparator across (and often within) tumor types is unlikely. Due to regulators’ lack of guidance on data collection and inconsistencies in patient-reported outcomes (PRO) collection, outcomes reported are highly variable.^11^

#### Regulatory assessment

Heterogeneity in patient characteristics, eg, tumor profile, location, and severity, causes variations in responses to PO and TA medicines, which drives the uncertainty related to the demonstration of relative effectiveness and makes regulatory assessment difficult.^10^

Inconsistencies in trial data collection may worsen problems.^3^ Furthermore, not all biomarkers are prognostic or predictive, and the relationship between biomarker-based or other surrogate endpoints and overall survival may be unclear.^13^

#### HTA process

HTA refers to the assessment of the value of a health technology in clinical and economic terms and is often required for reimbursement decisions. The differences between regulatory and HTA evidence expectations are particularly pronounced for PO medicines due to the evidence-generation challenges described. HTA requires comparative effectiveness and (in some countries) cost-effectiveness, for which head-to-head RCT evidence is most compelling. Some HTA bodies are unwilling to fully accept evidence from other sources, such as indirect comparisons or matched comparisons with historical controls, despite rigorous methods for doing so.^14^ For example, Germany restricts single-arm trials and favors overall survival as an efficacy endpoint.^15^ This issue is especially noticeable for TA medicines, which may have multiple indications with different standards of care, costs, and outcomes. In such cases, pooled estimates of clinical efficacy or cost-effectiveness for the intervention versus a basket of comparators may be difficult to interpret. Moreover, the relatively high budget impact of innovative treatments has been widely discussed in the literature.^16^

Precision oncology therapies require biomarker testing, eg, NGS or immunohistochemistry (IHC). In economic evaluations, the diagnostic test price may disadvantage a novel first-in-class therapy. Embedding molecular testing into health care infrastructure would reduce this cost for subsequent therapies.^2^ It is unclear, however, who should pay for these diagnostics, especially while therapies are still in trials.^17^ There is substantial geographic variation in NGS access between and within countries,^18^ and the new and stricter EU In Vitro Diagnostics Regulation may further delay NGS access and uptake.^18^

#### Payment and adoption

Beyond HTA, PO therapies also face payment and adoption issues, mostly due to divergent uncertainty-management processes and systems, such as conditional payment models.^2^ Moreover, several countries cannot or will not pay differentially for treatment indications, which could inhibit access in some indications if the fixed price does not offer value for money. Alternatively, differential net discounts are permitted in some countries, or a blended price may capture value across multiple indications.^19^ For TA drugs, inflexible single prices are unlikely to reflect value across all tumor types.

Concern was shared around the (often heavily discounted) prices used for early access schemes, with fears that this may impact the benchmark price when the drug receives approval, potentially disincentivizing participation by drug companies. Furthermore, the risk of low prices in early access schemes meaning manufacturers are not able to recoup the cost of single-administration therapies should be recognized as a potential barrier for this group of therapies, in cases where upfront costs for the provider are high and patient benefit may be realized in the long-term.

Real-world data (RWD) provide essential additional clinical evidence for real-world patient populations. However, RWD collection is fragmented across and within countries, and more clarity and agreement on how to best incorporate RWD in HTA evidence submissions is needed.^20^

### A vision for improved patient access to PO

**Figure 1.**
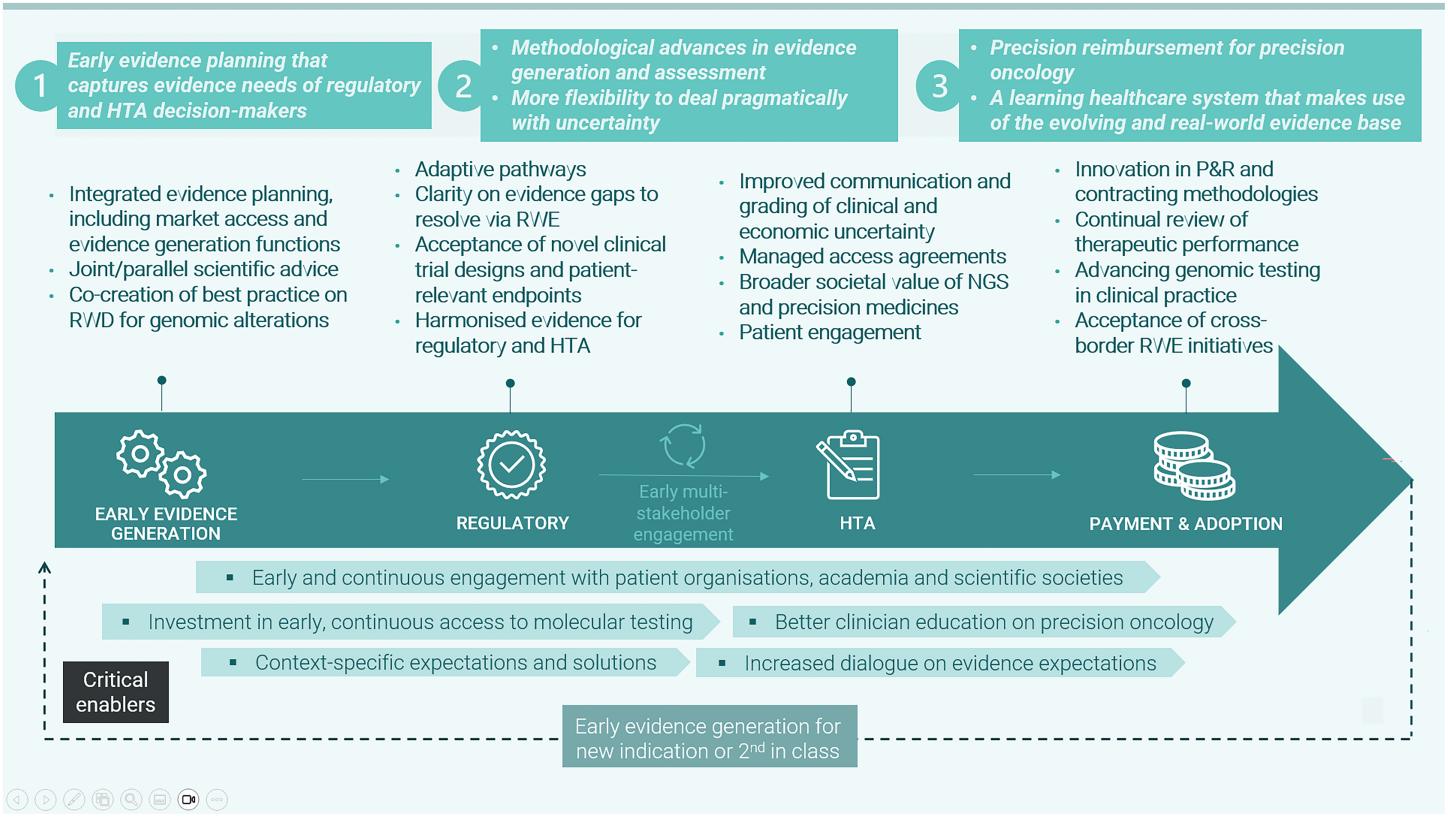
Vision for life-cycle assessment of PO medicines.

Precision oncology and TA therapies face many of the same issues as other cutting-edge technologies. We summarize several ways to improve their assessment and access. Figure 1 presents key actions and critical enablers throughout their life cycle.

This “life-cycle assessment” of PO medicines promotes dialogue and interaction between development, assessment, and access stages, supporting a learning health care system.^4,21^

#### Critical enablers

Many of the solutions require specific measures to be embedded throughout the development and assessment. These critical enablers include comprehensive biomarker testing, advanced clinician education, context-specific evidence dialogue, and continuous engagement with key stakeholders, especially patients.

Investing in early, continuous comprehensive testing has benefits beyond any specific therapy. Genomic screening is an investment in health infrastructure and population data and helps identify the right intervention for the right patient at the right time.^22^

Advanced education would help clinicians to better harness technological advances in oncology to improve patient outcomes and limit wasted time and resources on suboptimal care. In addition, academic and scientific societies could provide input, buy-in, and leadership to shape clinical guidelines.^20^ Moreover, to understand PO therapy opportunities, payers should be involved in ongoing education and up-to-date training programs on heterogeneity related to TAs and the value of NGS.

According to their mandates, regulatory, HTA, and payer organizations have different evidence expectations. Due to operational challenges and slow data maturation, RCT data may not be feasible as gold standard evidence for TA medicines. Early discussions should take place between manufacturer, regulators, HTA bodies, and other stakeholders on clinical development plans and evidence generation and should focus on the rationale for designing a clinical trial in a certain way, eg, as per Joint Scientific Consultations.

Another critical enabler is data infrastructure and governance investments to optimize RWD collection. This is crucial to resolve uncertainty about innovative medicines and gather data for managed access agreements (MAAs).

Finally, early, continuous engagement with patients and patient representatives is essential to build the patient voice into each stage and capture patient-relevant outcomes.

#### Life-cycle solutions

##### Early evidence generation

Joint/parallel scientific advice can engage industry, regulators, HTA agencies, and patient representatives earlier. Clinical trial designs should incorporate this advice to reduce post hoc analyses. Pharmaceutical companies should improve communication between development teams, market access, and RWE functions to collect PROs and economic or resource usage data. Patient organizations can help trials capture patient-relevant endpoints and also support by generating data on patient preferences and articulating unmet needs.

Stakeholder agreement on best practices for RWD collection and use as evidence (RWE) to inform decisions could also optimize early evidence-generation plans. One opportunity is using previously collected RWD to create synthetic matched controls. Since the use of RWD is still relatively new, it may take time to develop stakeholder consensus on the best sources of data that meet payer needs.

##### Regulatory assessment and HTA process

Increased dialogue and better collaboration between regulatory and HTA agencies on their respective evidence requirements would better align stakeholders’ expectations for measuring health outcomes, and thereby reduce data gaps in industries’ evidence-generation plans. To allow for greater flexibility and pragmatism in uncertainty considerations, regulatory and HTA assessment should promote methodological advances in evidence generation and assessment.

Improved coordination of evidence expectations across countries is also important. The recent EU HTA Regulation introduces Joint Clinical Assessments (JCA) and Joint Scientific Consultations on clinical aspects like safety and effectiveness and advises manufacturers on evidence requirements and study designs.^23^ The European Network for Health Technology Assessment (EUnetHTA) has produced a series of papers to inform the process.^24^

Iterative assessment and approval, eg, through an adaptive pathway approach,^25^ could facilitate market access while more data are collected. Versions of this approach have been successful for rare disease medicines, eg, the French early access authorization (AAP) programme.^2,26^ Nevertheless, the growing number of PO therapies requires more systemic solutions.

Regulatory assessment could suggest areas where RWE could fill gaps where RCTs are not feasible or ethical, and HTA agencies could be more open to indirect comparisons, validated surrogate endpoints (although it is recognized that these can be challenging for these therapies), and expert elicitation. Notably, some countries already actively promote RWE: The Cancer Drugs Fund (CDF) in England supports RWD collection to inform reassessments,^27^ and the National Institute of Health and Care Excellence (NICE) recently published a framework that outlines how RWD can fill evidence gaps. In practice, the CDF has offered the opportunity for 2 TA drugs—entrectinib and larotrectinib—to collect further RWD to resolve uncertainties. Data sources being utilized to fill evidence gaps include the Systemic Anti-Cancer Therapy (SACT) database, Public Health England molecular dataset, NHS England’s Blueteq data, Genomic England data as well as international datasets such as US data from Flatiron, and the European EUROCAN registries.^28,29^ In Italy, national registries have supported managed entry agreements through RWD collection for many years, which have shortened the time to patient access.^30,31^

##### Payment and adoption

Solutions at this stage should help in identifying innovative payment models for novel therapies and fostering a learning health care system that uses RWE.

Indication-specific or outcomes-based payments could improve patient access, payer value, and industry R&D incentives if health systems were more open to innovation in pricing, reimbursement, and contracting.^19^ Post-market data collection can help payers evaluate therapeutic performance and make appropriate (dis)investment decisions.^32^ Cross-border RWE initiatives could also encourage learning from other systems.^4^

## Conclusion: our call to action

This paper presents a holistic approach to improving PO medicine access and value throughout its life cycle, considering the challenges faced by these medicines. Working toward these solutions to maximize PO medicine value is a shared responsibility and benefits all stakeholders.

**Figure 2.**
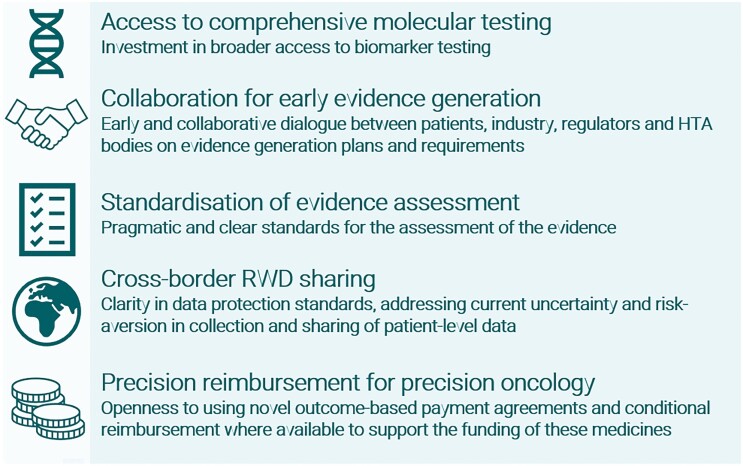
Call to action: our key recommendations.

Our call to action consists of 5 recommendations across the medicine life cycle. Figure 2 summarizes how these can be implemented.

### Access to comprehensive molecular testing

Molecular diagnosis is the only way to identify patients who could benefit from PO and TA therapies. Broader access to comprehensive molecular testing, such as NGS, is needed to overcome testing barriers and unlock the potential savings of identifying the right treatment at the right time for each patient.

NGS adoption is rising worldwide, although the pace varies between countries. Genomic testing is integrated into the cancer care pathway in France, with a wide geographic distribution of testing centers,^33^ although some argue that the funding model (a Ministry of Health grant) is insufficient and unsustainable. The UK has invested heavily in genomic sequencing and created genomic laboratory hubs to improve NGS networks: 52% of UK labs offer NGS, compared to 17% in the EU4.^3^ Despite NICE recognizing NGS’s value to the NHS, UK funding and access remain suboptimal. In Italy, the Ministry of Health has established a €5 million fund for NGS testing.^34^ NGS-capable lab networks are also needed. Based on the number and complexity of biomarkers and treatment availability, an Italian expert panel recommends limiting the clinical use of NGS to specific tumor types^35^

To expand molecular testing capabilities in countries with less experience, physicians could start with tumor types with the most genomic alterations and targeted therapies, such as lung cancer.

### Collaboration for early evidence generation

Early and collaborative dialogue between patient organizations, industry, regulators, and HTA bodies on evidence-generation plans and requirements would support a smoother pathway for these therapies that pragmatically consider generating the required evidence case-by-case.

In Belgium, national health authorities, academic centers, and industry engage in open dialogue, while in the UK and Canada, Scientific Advice programs by NICE and CADTH allow early feedback and HTA input into early evidence-generation plans.

### Standardization of evidence assessment

Pragmatic and clear standards for assessing evidence are needed, but currently, there is wide variation between countries. European guidance could improve alignment.

NICE’s RWE framework and Canada’s CADTH Guidance for Economic Evaluations of Tumor-Agnostic Products are good examples of sharing evidence collection standards.^36,37^ In addition, HAS recently published 6 “points of attention” that manufacturers should consider when submitting a dossier based on uncontrolled trial(s). These initiatives are welcome and should evolve as we learn from experiences with the datasets and methodologies, and co-create practical solutions.

### Cross-border RWD sharing

Clarity and uniformity in data protection standards interpretation would reduce uncertainty and risk-aversion in collecting and sharing patient-level data, enabling cross-border RWD sharing.

Initiatives such as the European Health Data Space,^38^ European Health Data Evidence Network,^39^ Data Analysis and Real World Interrogation Network,^40^ and the Get Real Initiative,^41^ in addition to RWE4Decisions^42^ should be leveraged to collect and share RWE.

### Precision reimbursement for PO

Health care systems should consider outcome-based and other novel payment arrangements to support access to PO. Supporting post-market evidence-based managed access pathways would support decision-makers to contribute to research and promote a learning health care system.

Examples of such approaches include England’s CDF which has provided interim access to 2 TA drugs, larotrectinib and entrectinib, while more data are collected to support reimbursement. In Italy, AIFA has recognized 2 TA drugs as fully innovative, allowing their reimbursement.^43^

## Discussion

Our call to action is to expand NGS access, foster a learning health care system, enable fast and equitable access to cost-effective treatments, and improve health outcomes.

While our calls to action are directed mostly at those developing, assessing, and providing access to medical care, patients must be at the heart of all of those actions. Patients must be empowered with the knowledge and information to discuss the best personalized treatment pathway for them, and patient groups must be involved across the development, validation, and implementation pathway of new PO strategies.^20^ Furthermore, patient organizations and charities can help disseminate advances in clinical practice and share critical insights.^20,44^

A learning health care system that encourages research and collaboration would maximize PO benefits. The “vision” proposes a more flexible and pragmatic approach to life-cycle assessment to better capture the value of medicines in regulatory, HTA, and reimbursement decisions. Many of our proposed policy positions could be applied to innovative medicines more broadly.

We recognize the complexity of the issues and the need for interim solutions, such as a stepped approach to expanding NGS testing. Furthermore, reassessments, new payment models, and performance-based outcomes require better data collection infrastructure.

The understanding of cancer has advanced, enabling more targeted and precise cancer treatment and care. Treatment assessment, reimbursement, and delivery must change to realize their potential. Our call to action intends to advance the dialogue and ongoing cooperation between all stakeholder communities to co-create solutions and cultivate a learning health system environment that improves health outcomes.

## Data Availability

No new data were generated or analyzed in support of this research.
